# Targeted Ablation of Distal Cerebrospinal Fluid-Contacting Nucleus Alleviates Renal Fibrosis in Chronic Kidney Disease

**DOI:** 10.3389/fphys.2018.01640

**Published:** 2018-11-21

**Authors:** Minzi Qiu, Jiawen Li, Lishan Tan, Mengbi Zhang, Guang Zhou, Tao Zeng, Aiqing Li

**Affiliations:** State Key Laboratory of Organ Failure Research, National Clinical Research Center for Kidney Disease, Nanfang Hospital, Southern Medical University, Guangzhou, China

**Keywords:** chronic kidney disease, dCSF-CNs, renin-angiotensin system, NADPH oxidase, sympathetic nervous system, inflammation, renal fibrosis

## Abstract

The potential function of distal cerebrospinal fluid-contacting nucleus (dCSF-CNs) in chronic kidney disease (CKD) development is poorly understood. We hypothesized that dCSF-CNs might affect the renin-angiotensin system (RAS) in kidney injury progression, with dCSF-CNs ablation potentially alleviating local RAS and renal fibrosis in rats after five-sixths nephrectomy (5/6Nx). Part of rats were randomly administered artificial cerebrospinal fluid (aCSF) intracerebroventricularly (icv), followed by 5/6Nx or sham operation; and other part of rats were administered Cholera toxin B subunit conjugated with saporin (CB-SAP) for dCSF-CNs lesion before 5/6Nx. The effect of CB-SAP on dCSF-CNs ablation was confirmed by double immunofluorescence staining. RAS component, NOX_2_ and c-fos levels in the subfornical organ (SFO), hypothalamic paraventricular nucleus (PVN) and hippocampus, as well as tyrosine hydroxylase (TH) and c-fos positive cells in rostral ventrolateral medulla (RVLM) were assessed. Next, the levels of RAS components (angiotensinogen [AGT], angiotensin-converting enzyme [ACE], Ang II type 1 receptor [AT1R], angiotensin-converting enzyme 2 [ACE2], and Mas receptor), NADPH oxidases (NOX_2_ and catalase), inflammatory cytokines (monocyte chemotactic protein 1 [MCP-1] and IL-6), and fibrotic factors (fibronectin and collagen I) were assessed. Less CB-labeled neurons were found in dCSF-CNs of CB-SAP-treated rats compared with 5/6Nx animals. Meanwhile, CB-SAP downregulated AGT, Ang II, AT1R, NOX_2_, catalase, MCP-1, IL-6, fibronectin, and collagen I, and upregulated ACE2 and Mas receptor, compared with CKD rats. More TH and c-fos positive cells were found in RVLM of 5/6Nx rats but the number decreased after dCSF-CNs ablation. Targeted dCSF-CNs ablation could alleviate renal inflammation and fibrosis in chronic kidney injury by inhibiting cerebral and renal RAS/NADPH oxidase.

## Introduction

The renin-angiotensin system (RAS), including the intrarenal (Zhuo et al., 1998) and cerebral (Burkhalter et al., 2001; Davisson, 2003; Wright and Harding, 2011) entities, is activated during progression of chronic kidney disease (CKD) (Crowley and Coffman, 2012; Cao et al., 2015; Villar-Cheda et al., 2017). Over-activity of the RAS results in kidney injury, leading to inflammation and renal fibrosis. Intracerebroventricular injection of angiotensin receptor blockers (ARBs) or antioxidants dramatically alleviates the intrarenal RAS, oxidative stress, and sympathetic nerve activity, resulting in the alleviation of renal fibrosis (Cao et al., 2015) and prevention of ongoing ischemia-reperfusion renal damage (Cao et al., 2017).

The interaction between the kidney and the brain is well established. Accumulating evidence indicates that nuclei, such as SFO, PVN, hippocampus, and RVLM, affect the activation of cerebral and renal RAS during disease progression in rats with CKD, which were administered high salt diet (Cao et al., 2015), as well as in mice with ischemia-reperfusion injury (Cao et al., 2017). Circulating Ang II binds to the angiotensin II type I receptor distributed in SFO and OVLT, which lacks blood-brain barrier, and induces a reaction cascade in cerebral RAS. Except for SFO and OVLT, recent studies have revealed a distinct type of neurons, termed distal cerebrospinal fluid-contacting nucleus (dCSF-CNs), which could also be a link between the cerebrospinal fluid and the brain. The neurons of dCSF-CNs are primarily distributed in the ventral periaqueductal gray of the brainstem and might be involved in signal transmission between the brain and the cerebrospinal fluid, since the soma of dCSF-CNs are located in the brain parenchyma; this process extends directly into the cerebrospinal fluid (Zhang et al., 2003), indicating that dCSF-CNs might be a sensor of chemicals in the cerebrospinal fluid. By transmitting and integrating the cerebrospinal fluid signals, the dCSF-CNs plays an important role in neuropathic pain and sodium homeostasis (Liu et al., 2014; Xing et al., 2015).

According to the specific distribution of dCSF-CNs, we hypothesized that it exerts a signal transmission effect on the reaction cascade of RAS between the cerebrospinal fluid and the brain during CKD progression. The aim of this study is to test the hypothesis that targeted ablation of dCSF-CNs via icv administration of Cholera toxin B subunit conjugated to saporin (CB-SAP), a cytotoxin, contributes to the inactivation of local RAS and thus the increase of renal inflammation and fibrosis in 5/6Nx rat models.

## Materials and Methods

### Animals

Male Sprague-Dawley rats (250 ± 50 g) purchased from the Nanfang Hospital Animal Experiment Center were maintained in a pathogen-free facility under temperature- and light-controlled conditions (24 ± 2°C, 12-h dark/light cycle) and 55 ± 5% humidity for at least 1 week prior to the experiments. All the experimental protocols were approved by the Animals Experiment Ethics Committee of Southern Medical University, Guangzhou, China.

### Drug Treatment

Cholera toxin B subunit conjugated to horseradish peroxidase (CB-HRP) for neural tracing was purchased from Thermo Fisher Scientific (Waltham, MA, United States), and CB-SAP was purchased from Advanced Targeting System (San Diego, CA, United States). The drugs were dissolved in artificial cerebrospinal fluid (aCSF).

### Intracerebroventricular Injection

Rats were anesthetized with sodium pentobarbital (40 mg/kg, i.p.) and immobilized by a digital stereotaxic instrument (Bilaney Consultants Ltd., Germany). Next, a midline incision was made followed by drilling a hole in the skull with a dental drill to insert a microinjection needle into the target site. The stereotaxic coordinates of the rat’s right lateral ventricles (LVs) were as follows: -1.2 ± 0.4 mm caudal to the bregma, 3.2 ± 0.4 mm ventral to the skull surface, and 1.4 ± 0.2 mm right of the median sagittal plane (George and Charles, 2005). Three microliters of CB-SAP (500 ng) and CB-HRP (900 ng) were administered slowly into the lateral ventricles (LVs) of normal rats over a 3-min period using a 5-μl Hamilton microsyringe equipped with a 32-gauge needle, which was then left in place for a further 7 min.

### Five-Sixths Nephrectomy (5/6Nx)

Five-sixths nephrectomy (5/6Nx) using a two-step procedure was performed in rats 14 days after CB-SAP administration by intracerebroventricular injection, as previously described (Chow et al., 2003; Liu et al., 2014). Rats were treated with sodium pentobarbital (40 mg/kg, i.p.). The upper and lower poles of their left kidney were then resected, followed by unilateral right nephrectomy 1 week later.

### Double-Staining Immunofluorescence

Localization of dCSF-CNs and the effect of targeted ablation of dCSF-CNs by icv administration of CB-SAP were assessed by double-staining immunofluorescence. Anti-CB (Abcam) and anti-NeuN (Santa Cruz Biotechnology) primary antibodies were used to treat sections of dCSF-CNs (4 μm) overnight at 4°C. The sections were then incubated with secondary antibodies for 1 h, washed, and visualized under a Zeiss microscope.

### Histology and Immunohistochemistry (IHC)

One-micrometer-thick sections of kidney samples were stained with hematoxylin and eosin (H&E), periodic acid-Schiff (PAS), and Sirius red, respectively. For IHC, four-micrometer-thick sections of kidney samples and specific brain nuclei of rats were sequentially incubated with anti-AGT (Swant), anti-ACE (Abcam), anti-AT1R (Abcam), anti-fibronectin (Sigma), anti-collagen I (Abcam), anti-Ang II (Peninsula Laboratories), anti-c-fos (Santa Cruz), and anti-tyrosine hydroxylase (BOSTER) primary antibodies overnight at 4°C, and secondary antibodies for 1 h. Next, c-fos-positive and TH-expressing neurons in RVLM were counted after double-staining with rabbit anti-rat c-fos and mouse anti-rat TH antibodies, as described previously (Cao et al., 2015).

### Western Blot

Proteins were extracted from kidney and brain nuclei tissue samples, and separated using 10% SDS–PAGE. The protein expression levels of RAS components, catalase, NOX_2_, MCP-1, IL-6, fibronectin, and collagen I in the kidney cortex and specific brain nuclei were determined with anti-AGT (IBL), anti-ACE (Abcam), anti-ACE2 (Abcam), anti-Mas receptor (Alomone Labs), anti-NOX_2_ (Santa Cruz Biotechnology), anti-catalase (Abcam), anti-MCP-1 (Abcam), anti-IL-6 (Abcam), anti-fibronectin (Sigma), anti-collagen I (BOSTER), and anti-β-actin (Cell Signaling Technology) primary antibodies. The membranes were then incubated with secondary antibodies for 1 h, washed, and analyzed.

### Real-Time PCR

After sacrifice, the kidney and specific brain nuclei were removed from rats and treated with TRIzol (Invitrogen, China) for total RNA extraction. After reverse transcription, real-time PCR with SYBR Green mix was performed using primers specific for rat, AT1R and glyceraldehyde-3-phosphate dehydrogenase (GAPDH) (Table 1).

**Table 1:** Real-time PCR primer sequences.

Gene	Forward	Reverse
AT1R	CAGTGTGCGCGTTTCATTATG	TGGTAAGGCCCAGCCCTAT
GAPDH	TGCCAAGTATGATGACATCAAGAA	AGCCCAGGATGCCCTTTAGT


### Data Analysis

Data are expressed as mean ± SEM (*n* = 6). Continuous variables among three groups were compared by one-way analysis of variance (one-way ANOVA). Statistical analyses were performed with SPSS 20.0 for Windows. *P* < 0.05 was considered statistically significant.

## Results

### Confirmation of Targeted Ablation of dCSF-CNs by icv Administration of CB-SAP

**FIGURE 1:**
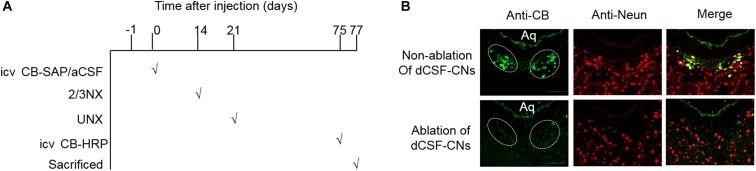
Schematic illustration of CB treatment and confirmation. **(A)** Schematic time-line of the CB treatment experiment. **(B)** Representative images of dCSF-CNs in the brainstem by CB-immunoreactive immunofluorescence after an intracerebroventricular injection of CB-SAP (magnification ×200; scale bar = 100 μm). CB (green) double-staining with the antibody against CB and the antibody-recognized Neun (red). Aq, ventral mesencephalic aqueduct; 2/3Nx, two-third of the left kidney was removed by surgical resection; UNX, unilateral nephrectomy.

To assess the role of dCSF-CNs in the RAS and oxidation stress regulation in CKD rats, targeted ablation of dCSF-CNs was performed by treatment with CB-SAP. CB-SAP is a cytotoxin that is coupled to Cholera toxin B subunit and specifically binds to dCSF-CNs neurons, leading to cell internalization and death (Liu et al., 2014; Xing et al., 2015). CB-SAP (500 ng/3 μl) and CB-HRP (900 ng/3 μl) Figure 1A for dCSF-CNs lesion and neural tracing, respectively, were injected into the lateral ventricles of rats, and the CB-labeled cells of dCSF-CNs were assessed under a Zeiss microscope. As shown in Figure 1B, CB-labeled cells of dCSF-CNs in the brainstem disappeared completely at 14 days after icv administration of CB-SAP.

### RAS Components Are Downregulated in Rats With dCSF-CNs-Lesions

**FIGURE 2:**
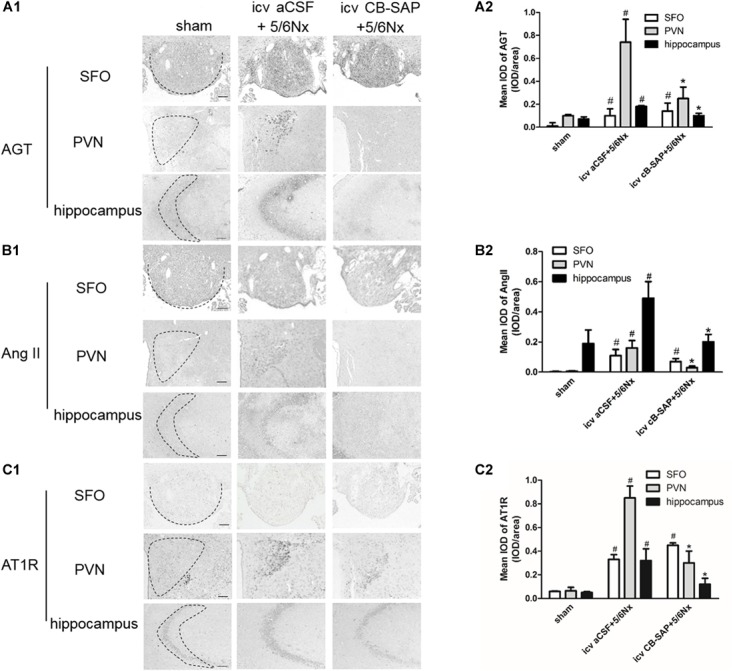
Overexpression of the central RAS in 5/6Nx rats. Representative photographs of immunohistochemistry staining of AGT **(A1)**, Ang II **(B1)**, and AT1R **(C1)** in SFO, PVN, and hippocampus, respectively [**(A1–C1)** magnification ×100; scale bar = 100 μm]. Semiquantitative data of AGT **(A2)**, Ang II **(B2)**, and AT1R **(C2)**. ^#^*P* < 0.05 versus sham in the respective group. ^∗^*P* < 0.05 versus icv aCSF+5/6Nx in the respective group.

The major components of the RAS are expressed in the brain (McKinley et al., 2003). To assess the role of dCSF-CNs in the activation of cerebral RAS, immunohistochemistry was performed to detect AGT, Ang II, and AT1R in the SFO, PVN, and CA3 region of the hippocampus (Figures 2A–C). The results showed strong immunoreactive signals for AGT, Ang II, and AT1R in the PVN and CA3 region of the hippocampus, but not in the SFO, in 5/6Nx rats without dCSF-CNs ablation. In comparison, nuclei of the PVN and CA3 region of hippocampal neurons from dCSF-CNs injured rats by icv administration of CB-SAP exhibited weak immunoreactivity for AGT, Ang II, and AT1R in the animals.

### CB-SAP Treatment Could Reduce c-fos Expression in the PVN and Hippocampus, as Well as TH and c-fos Expression Levels in RVLM

**FIGURE 3:**
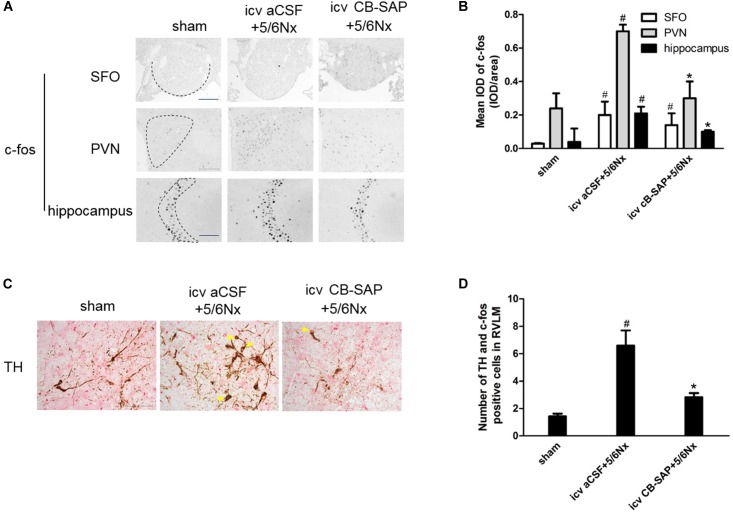
Expression of central TH and c-fos are increased in 5/6Nx rats. Representative photographs of c-fos in SFO, PVN and hippocampus **(A)**, and semiquantitative data **(B)**. Double labeling of TH (brown cytoplasmic) and c-fos (red nuclear) staining in RVLM **(C)**. Arrows indicate the TH- and c-fos positive cells in RVLM. The semiquantitative data are expressed as the mean ± SEM **(D)**. **(A)** Magnification ×100; scale bar = 100 μm. **(C)** Magnification ×200; scale bar = 100 μm. ^#^*P* < 0.05 versus sham in the respective group. ^∗^*P* < 0.05 versus icv aCSF+5/6Nx in the respective group.

The expression levels of c-fos and TH were increased in rats with CKD, suggesting increased central sympathetic drive. Targeted ablation of dCSF-CNs reduced c-fos expression in the nuclei of the PVN and CA3 region of hippocampal neurons but did not affect that of the SFO (Figures 3A,B). Significantly more c-fos-positive and TH-expressing neurons were found in RVLM as shown by double-labeling of TH and c-fos staining in 5/6Nx rats compared with the animals after dCSF-CNs damage (Figures 3C,D).

### Serum Creatinine, Blood Urea Nitrogen (BUN), and Urinary Albumin Levels Are Decreased in 5/6Nx Rats With dCSF-CNs Lesions

**FIGURE 4:**
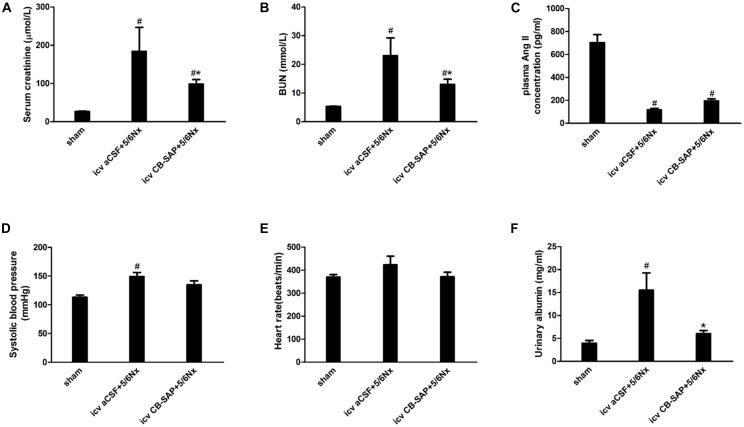
Physiological parameters of 5/6Nx rats after targeted ablation of dCSF-CNs. **(A)** Serum creatinine, **(B)** blood urea nitrogen (BUN), **(C)** plasma Ang II concentration, **(D)** systolic blood pressure (SBP), **(E)** heart rate, and **(F)** urinary albumin. Results are represented as mean ± SEM (*n* = 6). Data were analyzed using one-way ANOVA followed by least significant difference (LSD) test. ^#^*P* < 0.05 versus sham in the respective group. ^∗^*P* < 0.05 versus icv aCSF+5/6Nx in the respective group.

The establishment of 5/6Nx models was confirmed by significantly increased serum creatinine, BUN, and urinary albumin levels compared with control rats, and these amounts were significantly decreased in rats treated with CB-SAP for dCSF-CNs lesions (Figures 4A,B,F). There was no significant difference in the plasma Ang II concentration, systolic blood pressure (SBP), or heart rate between 5/6Nx rats without dCSF-CNs ablation and those with dCSF-CNs lesions (Figures 4C–E). However, there was a small drop (15.9 mmHg) in SBP in CKD-CB-SAP rats.

### Targeted Ablation of dCSF-CNs in the Brainstem of Rats Downregulates Intrarenal RAS Components

**FIGURE 5:**
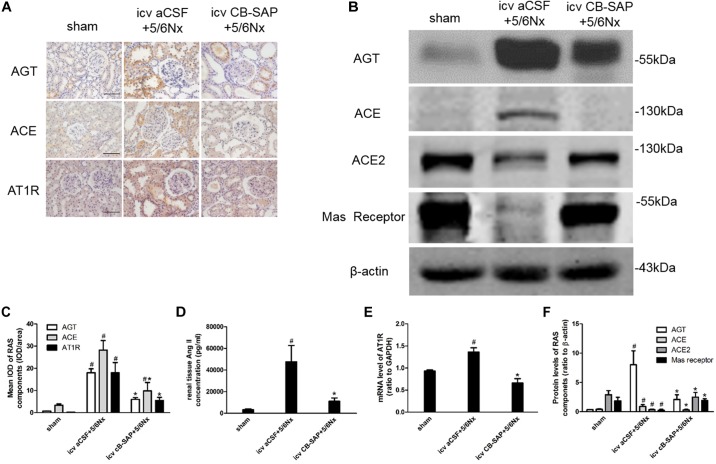
Overexpression of the renal RAS was inhibited by targeted ablation of dCSF-CNs. **(A)** Representative photographs of RAS component expression by immunohistochemistry (magnification ×200; scale bar = 100 μm). **(B)** Expression of RAS components detected by western blot. **(C)** Semiquantitative data of RAS expression by immunohistochemistry. **(D)** Renal tissue Ang II concentration. **(E)** AT1 receptor mRNA level analyzed by real-time PCR. **(F)** Semiquantitative data of RAS component expression by western blot. ^#^*P* < 0.05 versus sham in the respective group. ^∗^*P* < 0.05 versus icv aCSF+5/6Nx in the respective group.

There is a link between renal and cerebral RASs. Imbalance of the ACE/Ang II/AT1R and ACE2/Ang(1-7)/Mas receptor axes leads to RAS activation. Compared with CKD rats without dCSF-CNs lesions, the expression levels of AGT, in rats treated by CB-SAP showed reduced ACE and AT1R levels, while protein ACE2 and Mas receptor amounts were increased (Figures 5A–F). These data suggested that targeted ablation of dCSF-CNs might protect the kidney from RAS-activation-induced injury.

### Treatment With CB-SAP Reduces Renal NADPH Oxidase Levels and Inflammation in 5/6Nx Rats

**FIGURE 6:**
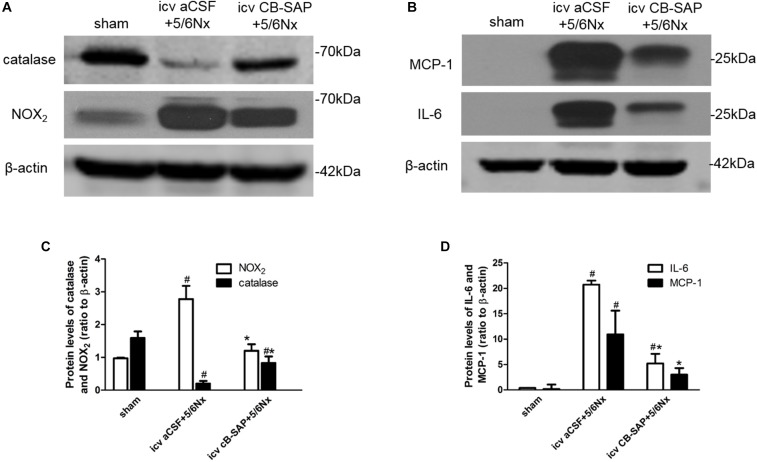
Expression of catalase, NOX_2_, and inflammatory cytokines in 5/6Nx rats. Representative photographs of protein levels of catalase and NOX_2_ **(A)** and inflammatory cytokines (MCP-1, IL-6) **(B)** in rats. Semiquantitative data of catalase and NOX_2_ **(C)**, as well as that of MCP-1 and IL-6 **(D)**. ^#^*P* < 0.05 versus sham in the respective group. ^∗^*P* < 0.05 versus icv aCSF+5/6Nx in the respective group.

To determine whether dCSF-CNs plays a role in renal NADPH oxidase production and inflammation induced by CKD, protein levels of NOX_2_, catalase, and inflammatory cytokines (MCP-1 and IL-6) were compared between 5/6Nx rats without dCSF-CNs ablation and the rats with dCSF-CNs lesions. The expression levels of catalase and NOX_2_ were significantly higher in 5/6Nx rats compared with control animals as well as rats treated with icv CB-SAP for dCSF-CNs ablation (Figures 6A,C). The protein levels of MCP-1 and IL-6 were higher in rats with CKD, but decreased in those treated with icv CB-SAP for dCSF-CNs ablation (Figures 6B,D).

### Ablation of dCSF-CNs by icv CB-SAP Could Attenuate Renal Fibrosis

**FIGURE 7:**
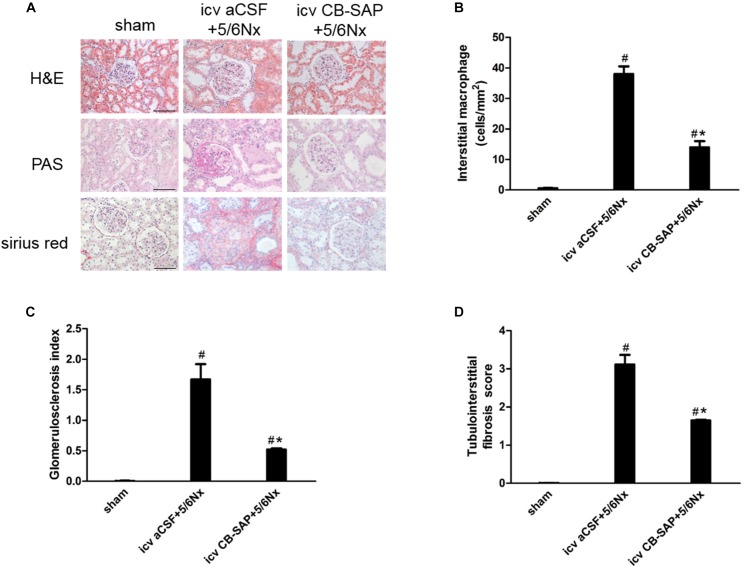
Renal inflammation and fibrosis in 5/6Nx rats was inhibited by targeted ablation of dCSF-CNs. **(A)** Representative photographs of renal fibrosis shown by H&E, PAS, and Sirius red-staining (magnification ×200; scale bar = 100 μm). Quantitative analysis of interstitial macrophage **(B)**, glomerulosclerosis index **(C)**, and tubulointerstitial fibrosis score **(D)**. ^#^*P* < 0.05 versus sham in the respective group. ^∗^*P* < 0.05 versus icv aCSF+5/6Nx in the respective group.

**FIGURE 8:**
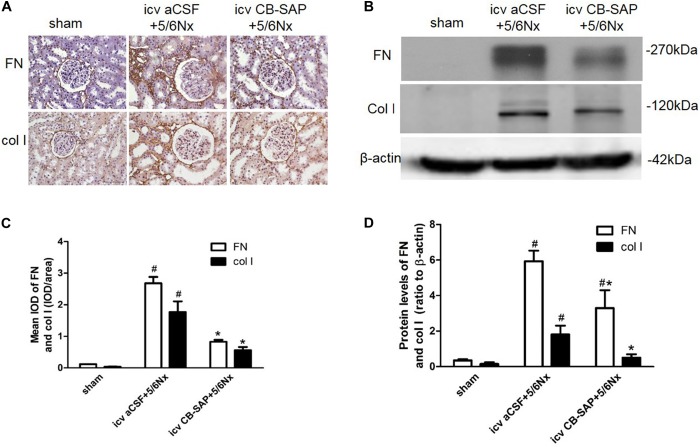
Expression of fibronectin and collagen I in 5/6Nx rats. Representative photographs of expression levels of fibronectin and collagen I in renal cortex **(A,B)**. **(A)** Magnification ×200; scale bar = 100 μm. Semiquantitative data of fibronectin and collagen I in renal cortex **(C,D)**. ^#^*P* < 0.05 versus sham in the respective group. ^∗^*P* < 0.05 versus icv aCSF+5/6Nx in the respective group.

To examine the effect of dCSF-CNs lesions on renal fibrosis, we evaluated the histological changes of the kidney tissue using H&E, PAS, and Sirius red-stained sections. Histological analysis showed that interstitial macrophage, glomerulosclerosis, and tubulointerstitial fibrosis in the kidney of 5/6Nx rats were more severe compared to sham rats, and these changes were alleviated through targeted ablation of dCSF-CNs (Figures 7A–D). Meanwhile, the expression levels of fibronectin and collagen I were increased in rats with CKD, but were decreased after icv administration of CB-SAP for dCSF-CNs ablation (Figures 8A–D). These findings indicated that treatment with CB-SAP for dCSF-CNs lesions provided protection against renal fibrosis in CKD rats.

## Discussion

Here, we report the interaction between kidney and dCSF-CNs in a rat model of CKD. The dCSF-CNs could act as a link between the cerebrospinal fluid and the brain for signal transmission in the development of kidney injury. Targeted ablation of dCSF-CNs decreased the expression levels of intrarenal and cerebral RAS components, as well as reactive oxygen species (ROS) production and the sympathetic drive, leading to renal protection and alleviated renal inflammation and fibrosis in CKD rats. To our knowledge, this is the first report showing that dCSF-CNs ablation prevents local RAS-activation-induced kidney injury.

Targeted ablation of dCSF-CNs by icv administration of CB-SAP, a cytotoxin, in rats was first established. The neural tracer CB-HRP was administered by intracerebroventricular injection 48 h before the rats were sacrificed. CB-HRP could not pass through the ependyma, and no CB-labeled cells were found in the SFO or OVLT that lack the blood-brain barrier, making it a reliable substance to trace dCSF-CNs (Xing et al., 2015). Immunofluorescent double-staining for CB and NeuN was used to determine the number of CB-labeled neurons. CB-positive cells in dCSF-CNs disappeared completely in the brainstem of rats at 14 days after CB-SAP injection, in line with previous findings (Liu et al., 2014). The dCSF-CNs is mainly described as a distinct neuron type whose soma is situated in the parenchyma of the brain with the axons extending into the cerebrospinal fluid in the ventricular cavity (Zhang et al., 2003). The CSF-CNs forms a part of circumventricular organs (CVOs) that lack the blood-brain barrier and acts as a sensor for the chemical composition of the cerebrospinal fluid (Vigh and Vigh-Teichmann, 1998). This specific structure makes dCSF-CNs serve as the link for information transmission between the cerebrospinal fluid and the brain. In addition, ultrastructural studies of dCSF-CNs indicated that these nuclei receive many axis-cylinder contacts from other brain regions, and in turn, project to other brain nuclei; this allows dCSF-CNs to play a role in signal transmission between the brain and the cerebrospinal fluid (Zhang et al., 2003). Therefore, dCSF-CNs ablation might, at least in part, interrupt signal transmission between the cerebrospinal fluid and brain. This indicates that treatment with CB-SAP for dCSF-CNs lesions in rats with CKD might be an effective means for investigating the signal transmission effect of dCSF-CNs on RAS activation during CKD progression.

Next, the expression levels of RAS components were assessed. As shown above, dCSF-CNs injury by icv administration of CB-SAP downregulated cerebral RAS components in the PVN and CA3 region of the hippocampus, but not in the SFO, as well as intrarenal RAS components. The classical RAS plays a crucial endocrine role in the physio-pathophysiology of the renal and cardiovascular systems. RAS perturbation has been shown to be involved in the pathogenesis of renal fibrosis (Gironacci et al., 2014). Ang II is the primary RAS product in regulating physiological responses after binding to the AT1 receptor. Indeed, Ang II decrease is considered an effective means to attenuate the reaction cascade of the RAS. Levels of Ang II in renal, PVN, and hippocampal CA3 region were lower in rats treated with CB-SAP for dCSF-CNs lesions compared with CKD rats. Meanwhile, reduced serum creatinine, BUN, and urinary albumin were observed in dCSF-CNs-injured rats (Figures 4A,B,F). These findings revealed that activation of cerebral and renal RAS in CKD rats was dependent on dCSF-CNs. Treatment with CB-SAP for dCSF-CNs ablation protected the kidney from RAS-activation-induced injury likely by disturbing the signal transmission of Ang II. However, no significant change was observed in plasma Ang II concentration or systolic blood pressure (SBP) after CB-SAP treatment (Figures 4C,D). These data suggested that protection from kidney injury by dCSF-CNs ablation might be dependent upon local, but not systemic RAS.

The balance between the classical RAS pathway, i.e., the ACE/Ang II/AT1R axis, and the “non-classical” RAS pathway, i.e., the ACE2/Ang-(1-7)/Mas receptor axis, probably plays an important role in the pathogenesis of many ailments, especially kidney diseases (Gironacci et al., 2014). In this study, in addition to reduced AGT, Ang II, and AT1R expression levels, an increase of ACE2 and Mas receptor expression in dCSF-CNs-injured rats was also observed. This finding indicated that dCSF-CNs probably modulated the intrarenal RAS by activating the ACE2/Ang-(1-7)/Mas receptor axis. The ACE2/Ang-(1-7)/Mas receptor axis is considered a protective pathway (Gironacci et al., 2014), which counters the actions of stimulated ACE/Ang II/AT1R axis by reducing oxidative stress and attenuates kidney injury (Chappell, 2012). During heart failure, the balance between the ACE/Ang II/AT1 receptor and ACE2/Ang-(1-7)/Mas receptor axes, is also very important in the regulation of central sympathetic outflow and humoral coordination (Zucker et al., 2014).

It is well known that the SFO is one of the main circumventricular organs with a semitransparent blood-brain barrier (Noda, 2006; Garcia-Lecea et al., 2017); therefore, the SFO can sense the blood’s chemical composition directly, and could constitute a linkage between the peripheral and central RASs (Cao et al., 2015, 2017). Circulating Ang II binds to specific high-affinity AT1 receptors in CVOs and the SFO, for example activating the RAS in the blood-brain barrier integrated circumventricular organs and inducing ROS production, and sequentially triggering a process of sympatho-excitation via an Ang II-AT1R-dependent pathway; this results in excitatory effects on the brain (Wright and Harding, 2011; Xu and Li, 2015). Meanwhile, intracerebroventricular injection of ARBs/antioxidants inhibits cerebral and intrarenal RASs (Cao et al., 2015, 2017). Studies have indicated that Ang II directly injected into the PVN could mediate an increase in sympathetic outflow (Patel et al., 2011), which could be inhibited by an AT1 receptor antagonist (Cao et al., 2015). Neurons of the SFO using Ang II as neurotransmitter for signal transmission to the PVN, and further to RVLM after integration, playing an important role in the integration of sympathetic outflow within the brain (Wang et al., 2015). In this study, icv administration of CB-SAP did not affect the expression levels of RAS components and c-fos in the SFO, possibly due to local generation of Ang II, as reported previously (Wright and Harding, 2011).

The interaction between the sympathetic nervous and RASs has been established. Central Ang II triggers the development and processing of sympatho-excitation via an Ang II-AT1R-dependent pathway in chronic heart failure (Zucker et al., 2014; Xu and Li, 2015). Our recent study showed that activation of reno-cerebral RAS axes may contribute to renal fibrosis by affecting the afferent and efferent sympathetic nerves in the 5/6Nx rat model (Cao et al., 2015). Meanwhile, the number of cerebral TH and c-fos positive cells is increased significantly in RVLM, indicating increased central sympathetic drive in salt-sensitive CKD rats (Cao et al., 2015, 2017). As shown above, ablation of dCSF-CNs significantly decreased the number of cerebral TH and c-fos positive cells in RVLM in CKD rats. Altogether, reduced Ang II in the brain after dCSF-CNs ablation might inhibit sympatho-excitation.

**FIGURE 9:**
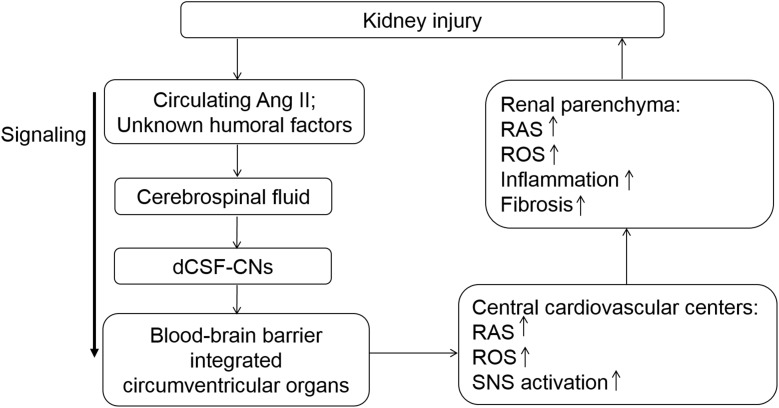
Schematic illustration of the modulation served by dCSF-CNs in kidney injury progression. The dCSF-CNs might exert a link between the cerebral fluid and the brain. The dCSF-CNs might act as the sensor of circulating Ang II and other unknown humoral factors in the cerebrospinal fluid, communicating signal to brain centers. The signal is then transferred from the cerebral fluid to the blood-brain barrier integrated circumventricular organs; this results in activation of cerebral- and renal-RAS/ROS axes consequently.

In conclusion, the present study elucidated the role of dCSF-CNs in the activation of intrarenal and cerebral RASs and renal injury progression (Figure 9). Targeted ablation of dCSF-CNs alleviated renal inflammation and fibrosis in CKD rats. These findings highlight the signal transmission effect of dCSF-CNs on the activation of local RAS, oxidative stress, and the sympathetic nervous system in CKD progression. The dCSF-CNs plays a critical role in the crosstalk between the kidney and the brain. Meanwhile, the dCSF-CNs lesion improved renal inflammation and fibrosis in CKD. This study unveiled a novel mechanism underlying chronic renal injury and the interaction between the brain and the kidney, therefore providing insights into novel therapeutic approaches such as dCSF-CNs ablation and central administration of RAS inhibitors.

## Ethics Statement

This study was carried out in accordance with the recommendations of Operational Guidelines for Ethics Committees that review biomedical research, 2000, World Health Organization. The protocols were approved by the Animals Experiment Ethics Committee of Southern Medical University, Guangzhou, China.

## Author Contributions

AL contributed to the design of the research. MQ conducted the experiment and drafted this manuscript. JL and LT provided help in removing the rats’ brain. MZ, GZ, and TZ provided help in removing the rats’ kidney. All authors have revised the intellectual content and the final version.

## Conflict of Interest Statement

The authors declare that the research was conducted in the absence of any commercial or financial relationships that could be construed as a potential conflict of interest.
